# Characteristics of Communities With High Rates of Hearing Difficulties and Falls: Findings From the RI and MA HADR

**DOI:** 10.1093/geroni/igaf122.080

**Published:** 2025-12-31

**Authors:** Eric Frimpong, Taylor Jansen, Josephine Boateng, Seokmin Kim, Yan-Jhu Su, Elizabeth Dugan

**Affiliations:** University of Massachusetts Boston, Boston, Massachusetts, United States; University of Massachusetts Boston, Boston, Massachusetts, United States; University of Massachusetts Boston, Dorchester, Massachusetts, United States; University of Massachusetts Boston, Quincy, Massachusetts, United States; The Pennsylvania State University, University Park, Massachusetts, United States; University of Massachusetts Boston, Boston, Massachusetts, United States

## Abstract

The National Institutes of Health (NIH) reports that one in three older adults live with hearing loss. Not only do hearing difficulties impact one’s quality of life, but it also increases the risk of falls among older adults, primarily due to their impact on balance and spatial awareness. Falls are a major public health concern, a leading cause of injury-related deaths among older adults and contribute to loss of independence. This study aims to examine communities with high rates of falls and hearing loss to identify the associated risk factors among older residents. Data was drawn from the 2025 Massachusetts and Rhode Island Healthy Aging Data Reports (HADR), the American Community Survey (2018-2022), and the Medicare Beneficiary Summary File (2020-2021). Among the total 427 communities, the average rate of 65+ adults who fell in the past year was 28% (range: 18.47-49.18%), while 12.55% (range: 0-42.31%) reported hearing difficulties. Communities with high fall rates (N = 85; Mean: 32.43%) and hearing difficulties (Mean: 17.33%) reported higher rates of older adults living alone, experiencing independent living and self-care difficulties, receiving food stamps, living below the poverty line, and being dually eligible for Medicare and Medicaid. The findings further indicate that communities with high rates of falls and hearing loss are home to older residents with self-reported independent living difficulties, lower employment, socioeconomic statuses, and physical activity. These findings are essential to guide policymakers on where to implement evidence-based programming like Matter of Balance and Tai Chi.

